# Clotrimazole‐loaded PLGA microparticles for local drug delivery to the vagina: Shape does matter

**DOI:** 10.1002/ame2.70198

**Published:** 2026-04-06

**Authors:** Yanyu Li, Jingting Luo, Shitao Zou, Xin Zhang, Zhen Wang, Ziwen Jiang, Jingjie Wang, Zhaoxia Liu, Zhimin Zhou

**Affiliations:** ^1^ Hubei Key Laboratory of Natural Products Research and Development, College of Biological and Pharmaceutical Sciences China Three Gorges University Yichang China; ^2^ College of Biological and Pharmaceutical Sciences China Three Gorges University Yichang China; ^3^ Biomedical Barriers Research Center Institute of Biomedical Engineering, Chinese Academy of Medical Sciences and Peking Union Medical College Tianjin China; ^4^ Department of Gynecology, Beijing Obstetrics and Gynecology Hospital Capital Medical University, Beijing Maternal and Child Health Care Hospital Beijing China; ^5^ State Key Laboratory of Eye Health, Eye Hospital Wenzhou Medical University Wenzhou China

**Keywords:** clotrimazole, pharmacokinetics, PLGA microparticles, shape effect, vaginal drug delivery

## Abstract

**Background:**

Intravaginal drug administration is the most promising strategy for treating vaginal infections to protect women's health, such as vulvovaginal candidiasis (VVC). However, conventional vaginal formulations in the clinic face challenges such as drug leakage and low bioavailability due to the mucus barrier and vaginal self‐cleaning behavior. To address these limitations, we designed and optimized clotrimazole‐loaded PLGA non‐spherical microparticles (CPNMs) for vaginal drug delivery.

**Methods:**

CPNMs and clotrimazole‐loaded PLGA porous microspheres (CPMs) were prepared by the double emulsion‐solvent evaporation method. The physicochemical features and in vitro drug release kinetics were characterized, and the effect of shape on drug delivery were also investigated. After intravaginal administration of CPMs and CPNMs, the pharmacokinetics and biocompatibility in the vagina of mice were studied.

**Results:**

Both CPMs and CPNMs exhibited sustained release features in vitro. Initially, there was a rapid release within the first 24 h and the cumulative release of CPMs and CPNMs was 46.55% and 56.89% over 7 days, respectively. Compared to CPMs, CPNMs demonstrated an extended drug release in vivo, maintaining a residence duration of at least 72 h, which was beneficial for clotrimazole delivery at a high concentration in the vagina of mice. Histological evaluation demonstrated the low cytotoxicity and good biocompatibility of CPNMs, with no obvious damage to vaginal epithelial cells.

**Conclusion:**

This vaginal drug delivery system offers potential applications for managing VVC, along with other health concerns in women.

## Graphical abstract

Clotrimazole‐loaded PLGA microparticles for intravaginal delivery.

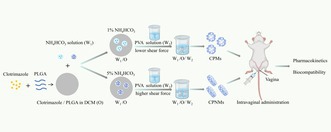

## 1 INTRODUCTION

Vulvovaginal candidiasis (VVC) is a fungal infection of the vaginal mucosa caused by *Candida* species, primarily associated with factors such as extensive use of antibiotics and hormonal medications, pregnancy, diabetes mellitus, and unprotected sexual intercourse.^1^ VVC affects 75% of women at least once during their lifetime and it can easily recur and has a severe adverse impact on women's health.^2^, ^3^, ^4^ Currently, clinical treatment of VVC involves oral administration or injection of various antifungal drugs, including clotrimazole (CLZ), fluconazole, amphotericin B, nystatin, and miconazole.^5^ Compared with systemic administration, intravaginal drug administration using suppositories, tablets, creams or gels is an alternative strategy aligned with the specialized physiological structures of women's reproductive system, which can avoid the first‐pass effect and reduce systemic toxicity.^4^, ^5^, ^6^, ^7^ Nevertheless, frequent administration is required for the above‐mentioned conventional formulations owing to insufficient retention in the vaginal cavity and difficulty reaching the epithelial surface due to poor penetration of the mucus barrier.^5^, ^8^, ^9^, ^10^ Therefore, it is important to exploit local drug delivery systems to enhance the patient compatibility and clinical effectiveness of VVC treatments.

Nowadays, vaginal drug delivery systems mainly comprise mucus penetrating approaches and mucoadhesive strategies.^5^, ^11^ Cervicovaginal mucus (CVM) can trap foreign particles through both adhesive and steric interactions (preventing the diffusion of particles exceeding 500 nm through the mucin mesh).^12^ Thus, by modulating particle size and surface characteristics, particle penetration across the mucus barrier for drug delivery to the vaginal epithelium can be facilitated.^11^, ^12^ Ensign et al.^13^ and Wang et al.^14^ developed mucus‐penetrating particles by modifying the particle surfaces with a poly(ethylene glycol) (PEG) coating that allowed the particles to reach the vaginal epithelial surface across the mucus barrier. Making the diameter of particles smaller than the pores in CVM (average 340 ± 70 nm) allows them to be transported through the mucus deep into the vaginal rugae and ensures uniform distribution.^15^ Compared to spherical particles, flexible tubular formulations can penetrate faster and deeper in the mucus with improved bioavailability and therapeutic efficiency.^16^ In addition, hyaluronic acid‐coated Eudragit RL100 nanoparticles have proved to be a superior vaginal therapeutic alternative for treating VVC due to the sequential crossing of the mucosal barrier and cell membrane mediated by CD44 receptors.^17^ To counteract fluid absorption and reduce leakage, hypotonic formulations may further facilitate rapid penetration of drug molecules through the mucin mesh to the vaginal epithelium, and provide enhanced biocompatibility and better long‐term retention for improved vaginal delivery.^18^, ^19^ Mucoadhesive drug delivery systems, microparticles, fiber mats, emulsions, and gels can also extend drug retention times in the vagina and deliver the drugs from a reservoir to vaginal epithelium with sustained release profiles.^8^, ^18^, ^19^, ^20^, ^21^, ^22^ Microparticle‐based drug formulations, in particular, have distinct advantages, such as the maintenance of mucus structure and vaginal microenvironment due to the limiting effect of size of carrier materials on mucus penetration and cell uptake.^23^ Moreover, compared with nanoscale particles, the microparticles exhibit distinct advantages for practical applications in VVC treatment, including easy collection, superior drug loading capacity, prolonged sustained‐release duration, and excellent stability.^21^, ^24^ It should be noted here that, to date, spherical microparticles are still prevalent in vaginal drug delivery systems. Non‐spherical particles have attracted considerable attention in drug delivery owing to their specific biological responses, such as evading cellular phagocytosis, enhanced margination, prolonged drug retention time, and conspicuous placental barrier transportation.^16^, ^25^, ^26^, ^27^, ^28^ However, the mechanisms underlying the superiority of non‐spherical microparticles in vaginal drug delivery are not known.

CLZ is a broad‐spectrum azole antifungal agent and is recommended as a first‐line therapeutic agent for clinical treatment of VVC.^1^, ^29^ It can inhibit the formation of cell membranes and alter their permeability, leading to the death of fungal cells, exhibiting potent antifungal effects across diverse fungal strains including *Candida albicans*.^30^, ^31^ However, the limited aqueous solubility of CLZ (0.49 μg/mL) results in low bioavailability and poor therapeutic efficacy.^29^ Poly(lactic‐co‐glycolic acid) (PLGA) has attracted significant attention in drug delivery systems owing to its exceptional biodegradability and biocompatibility.^32^ In our previous studies, we prepared PLGA non‐spherical microparticles by the emulsion‐solvent evaporation method in the presence of sodium tripolyphosphate or ammonium bicarbonate.^33^, ^34^ In particular, PLGA disc‐like microparticles were transformed from porous microspheres via adjusting shear force and ammonium bicarbonate concentration for hydrophobic drug encapsulations such as simvastatin and lidocaine, which exhibit unique drug release kinetics in different release media.^27^, ^34^ More importantly, dexamethasone microcrystals combined with lidocaine‐loaded PLGA non‐spherical microparticles showed a significant synergistic pharmaceutical effect due to their prolonged duration in the tympanic cavity and sustained drug release from the reservoir across the barrier of the round window membrane.^27^ Therefore, we aimed to construct CLZ‐loaded PLGA non‐spherical microparticles and assess the biological responses to their intravaginal delivery as a potential VVC treatment.

In this study, we prepared CLZ‐loaded PLGA disc‐like non‐spherical microparticles (CPNMs), with the corresponding porous counterparts (CPMs) as controls, through the double emulsion‐solvent evaporation method via NH_4_HCO_3_ concentration optimization and shear force adjustment.^34^ Subsequently, the physicochemical features and in vitro release kinetics of CLZ in CPMs and CPNMs were characterized. Finally, after intravaginal administration in mice, the pharmacokinetics and biocompatibility of CPNMs and CPMs in the vagina were evaluated.

## 2 METHODS

### 2.1 Materials

Clotrimazole (CLZ) was obtained from Shanghai Macklin Biochemical Co., Ltd. (Shanghai, China). Poly(lactic‐co‐glycolic acid) (PLGA, Mw: 50 000; 50: 50) was purchased from Jinan Daigang Biomaterial Co., Ltd. (Shandong, P. R. China). Ammonium bicarbonate (NH_4_HCO_3_) was acquired from Shanghai Aladdin Biochemical Technology Co., Ltd. (Shanghai, China). Dichloromethane (DCM) originated from Tianjin Feng‐Chuan Chemical Reagent Co., Ltd. (Tianjin, China). Polyvinyl alcohol (PVA) with 88% hydrolysis degree and 500 polymerization degree was procured from Sinopec Sichuan Vinylon Works (Sichuan, China). The remaining chemical substances were analytical‐grade materials, acquired from various commercial sources and utilized in their original form without additional processing.

### 2.2 Preparation of CPMs and CPNMs

CPMs were prepared by the double emulsion‐solvent evaporation method (W_1_/O/W_2_) using NH_4_HCO_3_ as the porogen, according to our previous report.^34^ Briefly, 200 mg of PLGA and specified amounts of CLZ were dissolved in 8 mL of DCM as the oil phase (O) and 1.5 mL of 1% (w/v %) NH_4_HCO_3_ aqueous solution (W_1_) was added to the oil phase (O) to form the primary W_1_/O emulsion. The primary W_1_/O emulsion underwent homogenization with a homogenizer (IKA, Germany) operating at 3600 rpm for 2 min. Subsequently, the W_1_/O emulsion was quickly and evenly dispersed into 1% (w/v %) PVA (100 mL) aqueous solution (W_2_), then continuously stirred at 400 rpm overnight to completely evaporate the DCM. The prepared porous microspheres containing CLZ were collected, subjected to triple washing with purified water, and subsequently freeze‐dried for preservation. For the preparation of CPNMs, the concentration of NH_4_HCO_3_ needs to be increased to 5%. The primary W_1_/O emulsion homogenization speed was also increased but other steps were the same as for CPM preparation.

Blank PLGA porous microspheres (PMs) and blank PLGA non‐spherical particles (PNMs) containing no CLZ were also prepared.

### 2.3 Characterizations

#### 2.3.1 Scanning electron microscopy (SEM)

The surface morphology of raw CLZ, CPMs, CPNMs and the blank counterparts were characterized using SEM (ZEISS Sigma 360, SUPRA 55 V P). A diluted particle suspension was carefully dripped onto a clean silicon wafer and the water evaporated naturally at room temperature. Then, a thin gold film was sprayed onto the surface of the particles using an ion coater (ETD2000C). Subsequently, SEM was performed with an acceleration potential set to 3 kV. Quantitative measurements were obtained through the Nano Measurer application, randomly selecting 100 or more particles from the SEM images, and measuring their diameters. Then, the measured diameters were subjected to statistical analysis to calculate the average size of CPMs and CPNMs.

#### 2.3.2 Fourier transform infrared spectroscopy (FTIR)

Raw CLZ, PMs, PNMs, CPMs, CPNMs, a physical mixture of CLZ and PMs, and a physical mixture of CLZ and PNMs were mixed with KBr separately and then compressed into tablets. Subsequently, their IR spectra were recorded using the FTIR spectrometer (Nicolet iS10, USA) in the transmission mode.

#### 2.3.3 X‐ray diffraction analysis (XRD)

The crystalline properties of raw CLZ, PMs, PNMs, CPMs, CPNMs, a physical mixture of CLZ and PMs, and a physical mixture of CLZ and PNMs were analyzed using a Rigaku D/MAX‐2500 X‐ray diffractometer (Japan). Measurements were conducted with Cu Kα radiation (*λ* = 1.541 78 Å) across a 2θ angular range from 0° to 60°.

#### 2.3.4 Differential scanning calorimetry (DSC)

Thermal transitions including glass transition temperature (Tg), melting temperatures (Tm) and enthalpy changes (ΔHm) of raw CLZ, PMs, PNMs, CPMs, CPNMs, a physical mixture of CLZ and PMs, and a physical mixture of CLZ and PNMs were analyzed. Measurements were conducted using a DSC (200 F3, Netzsch, Germany) with precisely weighed 7 mg samples sealed in standard pans. The thermal protocol involved initial heating from 10 to 200°C, followed by a 5 min isothermal hold, controlled cooling under nitrogen purge (20 mL/min flow rate), and subsequent reheating to 200°C at 10°C/min.

#### 2.3.5 Drug loading and encapsulation efficiency

The quantification of CLZ loading and encapsulation efficiency in CPMs and CPNMs were determined employing UV spectrophotometric analysis (PerkinElmer Lambda 35). Precisely weighed 10 mg aliquots of CPMs and CPNMs were solubilized in DCM (10 mL), filtered through 0.22 μm membranes, and analyzed at 215 nm. The calculation formulas are presented below^34^:

$$\begin{array}{c} \text{Drug loading}=\left(\text{weight of the}\;\mathrm{CLZ}\;\text{in CPMs and CPNMs}/\right.\\ \left.\text{weight of CPMs and CPNMs}\right)\times 100\%, \end{array}$$

$$\begin{array}{c} \text{Encapsulation efficiency}\\ =\left(\text{drug loading}/\text{theoretical drug loading}\right)\times 100\%. \end{array}$$

#### 2.3.6 In vitro drug release kinetics

Specific weights of raw CLZ (1 mg/mL), CPMs and CPNMs (both 10 mg/mL) were transferred into a dialysis bag (MWCO 8000–14 000 Da), containing 2 mL of ethanol/PBS dissolution medium (30:70 v/v, pH = 4.5).^35^ The dialysis bag was then immersed in 38 mL of identical dissolution medium. The entire release system was incubated at 37°C and shaken at 100 rpm for 7 days. Continuous sampling was conducted for 7 days, with 1.0 mL of medium collected for each sample and an equal volume of fresh release medium replenished simultaneously. The concentration of CLZ in the samples was quantified using HPLC (Waters 2695, USA) at 215 nm. The mobile phase consisted of methanol and 0.05 mol/L potassium dihydrogen phosphate solution (70:30, pH = 5.7–5.8, adjusted with 10% phosphoric acid) at a flow rate of 1.0 mL/min. The calibration curve was generated and used to calculate the concentrations of CLZ. An excellent linearity was observed over the concentration range of 2.5–80 μg/mL (*R* ^2^ = 0.9996).

To investigate the morphological changes of CPMs and CPNMs after drug release over different time (at 0.5 h, 2 h, 1 day, 3 days, 5 days and 7 days), SEM characterization was performed.

### 2.4 Animals

For animal experiments, female BALB/c mice aged 6–8 weeks (weight range 18 ± 2 g) in good health condition were obtained from the Laboratory Animal Center at the Institute of Radiation Medicine, Chinese Academy of Medical Sciences (Tianjin). The experimental procedures involving these animals received formal approval from the Animal Ethical and Welfare Committee of the Experimental Animal Center of the Institution of Radiation Medicine, Chinese Academy of Medical Sciences (approval number: IRM/2‐IACUC‐2506‐033).

### 2.5 Local pharmacokinetics study

Female BALB/c mice in good health were randomly distributed across three experimental cohorts. Each cohort was intravaginally administered 20 μL of 1% sodium hyaluronate solution containing raw CLZ, CPMs, or CPNMs (CLZ concentration of 2.5 mg/kg).^19^, ^36^, ^37^ At intervals of 1, 2, 6, 8, 12, 24, 48, and 72 h following intravaginal administration, vaginal lavage was performed by rinsing with 50 μL of saline, then, the vaginal lavage fluid was collected and stored at −20°C. The samples were analyzed using HPLC, with chromatographic parameters aligned to match the in vitro dissolution protocol.

### 2.6 Biocompatibility study

#### 2.6.1 The in vitro blood compatibility

The hemolysis tests were conducted on raw CLZ, CPMs and CPNMs to determine their blood compatibility. In short, fresh blood from BALB/c mice was collected in EDTA‐coated vacuum containers, the supernatant was removed after centrifugation. The red cell pellet was washed 3–5 times with physiological saline and finally diluted with physiological saline (5% in saline, v/v). Using saline as the solvent, suspensions containing 0.94, 1.88, 3.75, 7.5, 15 and 30 μg/mL of CLZ in raw CLZ, CPMs, and CPNMs respectively, were prepared. Subsequently, the samples were maintained in 250 μL of diluted blood solution at 37°C for 3 h. Saline solution and distilled water served as the negative and positive reference groups respectively. Following centrifugation of the test samples, optical density readings of the supernatant were obtained at 540 nm using a microplate reader. The hemolytic percentage was determined through the following computational equation^38^:

$$\text{Hemolysis ratio}\;\left(\%\right)=\frac{{\mathrm{OD}}_{\text{sample}}-{\mathrm{OD}}_{\text{negative control}}}{{\mathrm{OD}}_{\text{positive control}}-{\mathrm{OD}}_{\text{negative control}}}\times 100\%.$$

OD_sample_, the optical density reading obtained from the sample group; OD_positive control_, the optical density reading obtained from the positive control; OD_negative control_, the optical density reading obtained from the negative control.

#### 2.6.2 Vaginal irritation test‌

Four groups of healthy female BALB/c mice (*n* = 6, each group) were randomly formed and subjected to intravaginal administration with 20 μL of PBS, 20 μL of 1% sodium hyaluronate solution containing raw CLZ, CPMs, or CPNMs at a dosage of 5 mg/kg CLZ daily for 7 uninterrupted days. During the entire drug administration period, the body weight of the test animals was recorded daily, and the external vaginal morphology was observed for hyperemia, erythema, purulent exudate, ulceration and other inflammations.^20^

#### 2.6.3 Biocompatibility assessment

Blood samples from mice were collected for hematological evaluation after continuous administration for 7 days. After intraperitoneal administration of 2.5% tribromoethanol (5 mL/kg), the mice were euthanized by cervical dislocation, and vaginal tissues as well as major organs (heart, liver, spleen, lung, and kidney) were collected. After being fixed in 10% formalin for 48 h, all the tissues were embedded and sliced for histological analysis after H&E (Solarbio, Beijing, China) staining. The vaginal tissue slices were also stained separately by PAS (Solarbio, Beijing, China).^20^

### 2.7 Statistical analysis

Statistical analysis was performed using SPSS version 27.0 (SPSS Inc., Armonk, NY, USA). Mean ± standard deviation (SD) is shown for all experimental data. Differences were analyzed by one‐way analysis of variance (ANOVA), with a subsequent Tukey's HSD post hoc test. Statistical significance was defined as *p* < 0.05.

## 3 RESULTS

### 3.1 Preparation of CPMs and CPNMs

**FIGURE 1:**
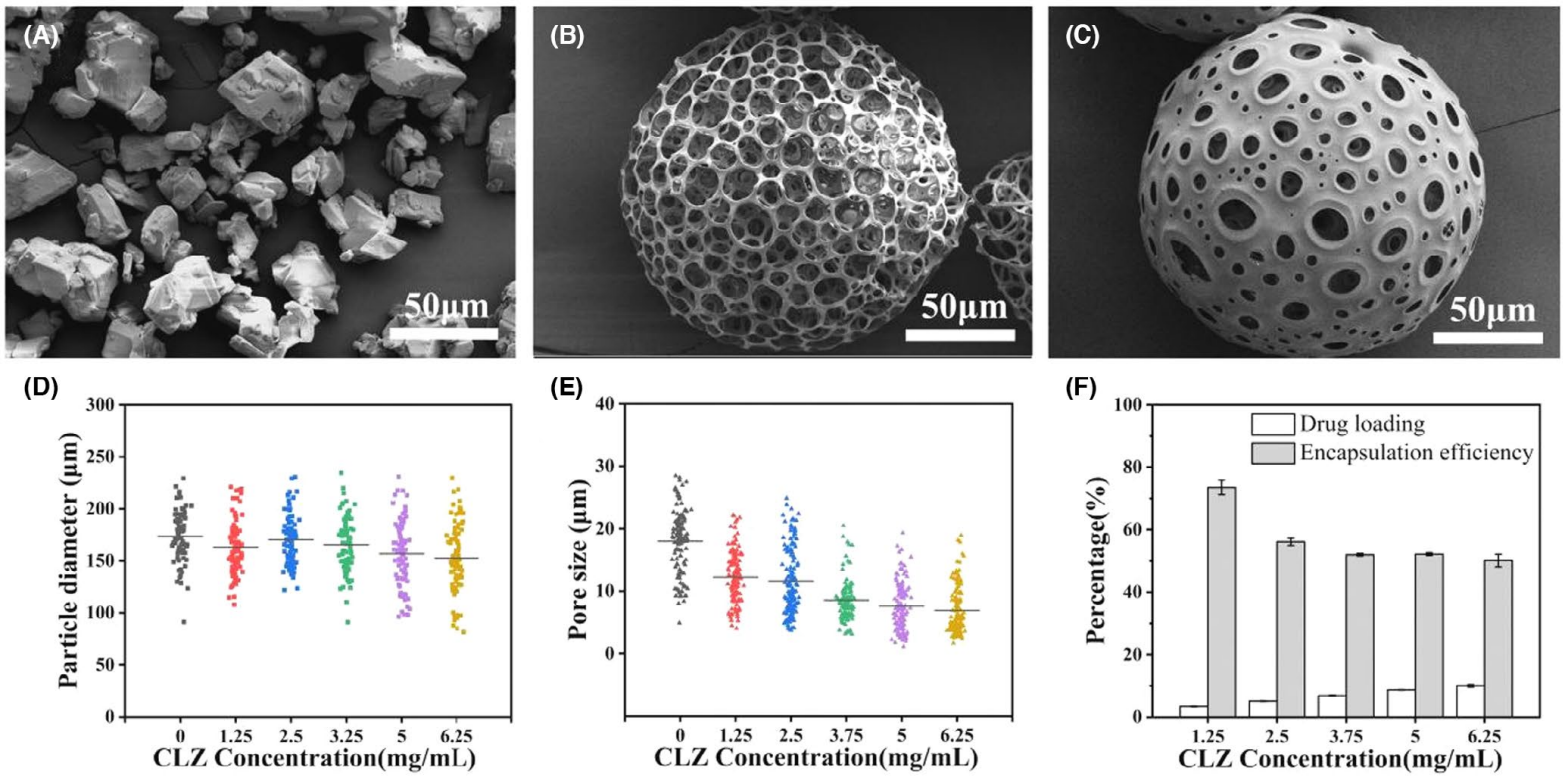
Physicochemical characterization of CPMs. SEM images of raw CLZ (A), PMs (B), CPMs (CLZ concentration: 6.25 mg/mL) (C), size distribution (D) and pore diameter distribution (E) of different CPMs with varying CLZ concentrations, drug loading and encapsulation efficiency of different CPMs with varying CLZ concentrations (F). Data are shown as mean ± SD, *n* = 3.

As shown in Figure 1A, the particles of raw CLZ are of uneven size and irregular shape. The prepared PLGA blank porous microspheres exhibited a smooth surface and an interconnected internal and external pore structure (Figure 1B). CPMs had a similar porous structure and size to PMs (Figures 1C and S1). With the increase of CLZ concentration from 1.25 to 6.25 mg/mL, the microspheres diameter of CPMs ranged from 91.27 to 229.21 μm, with an average diameter of 152.66 ± 32.14 μm (Figure 1D), which is consistent with our previous report.^39^, ^40^ The average pore diameter of CPMs was changed from 18.11 ± 4.90 μm to 6.93 ± 3.85 μm (Figure 1E).^41^ Meanwhile, the drug loading increased from 3.50% ± 0.11% to 10.03% ± 0.41%; however, the encapsulation efficiency decreased from 73.53% ± 2.31% to 50.13% ± 2.04% (Figure 1F).

**FIGURE 2:**
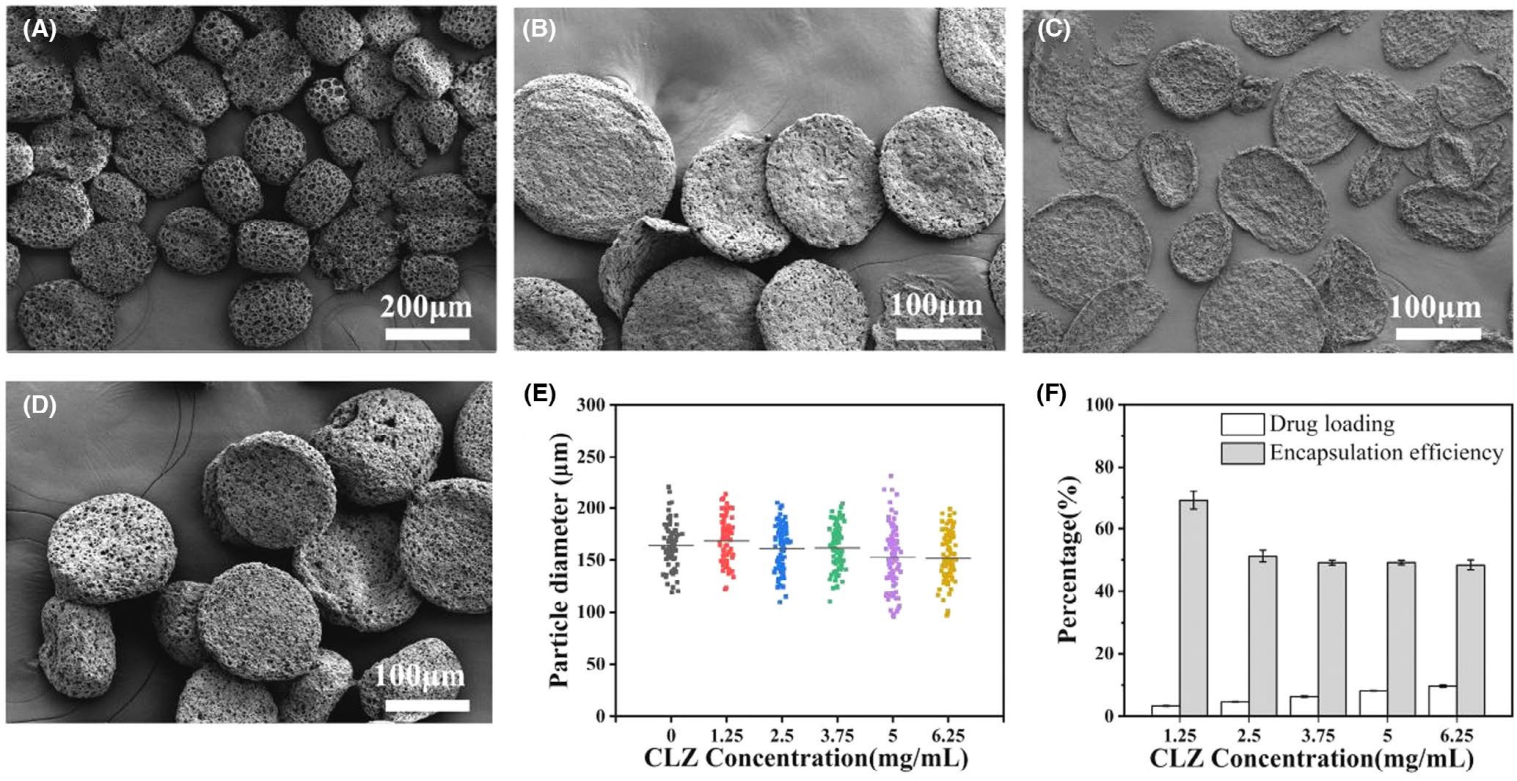
SEM images of PLGA non‐spherical microparticles under various parameters. (A) 5%, 3600 rpm; (B) 5%, 7200 rpm; (C) 5%, 10 800 rpm; (D) 5%, 7200 rpm, (CLZ concentration: 6.25 mg/mL). (E, F) Size distribution (E) and drug loading and encapsulation efficiency (F) of different CPNMs with varying CLZ concentrations. Data are shown as mean ± SD, *n* = 3.

With the increase of NH_4_HCO_3_ concentration to 5%, as shown in Figure 2A, we observed the partially collapsed structure of PLGA blank porous microspheres, resulting in the formation of non‐spherical particles, predominantly in the shape of discs. When the homogenization speed increased from 3600 to 7200 rpm, all the obtained particles were non‐spherical disc‐like particles with coarse surfaces (Figure 2B). With a further increase to 10 800 rpm, the obtained non‐spherical particles were thinner (Figure 2C). Therefore, 5% NH_4_HCO_3_ was finally chosen as the optimal porogen concentration and the stirring speed for W_1_/O homogenization was set at 7200 rpm for CLZ encapsulation (Figure 2D). We clearly obtained a high yield of CPNMs with coarse surfaces. With increasing CLZ concentration, the drug loading increased while the encapsulation efficiency decreased, which was consistent with the trend for CPMs (Figures 1D,F and 2E,F). The maximum drug loading was 9.67% ± 0.36%, and the encapsulation efficiency was 48.37% ± 1.57%, but both were slightly lower than for CPMs (Figures 1F and 2F). To avoid drug wastage and optimize drug delivery, CPMs and CPNMs were prepared with a CLZ concentration of 6.25 mg/mL in further experiments.

**FIGURE 3:**
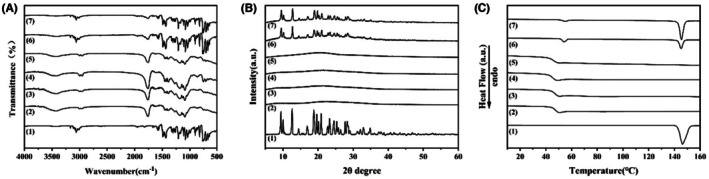
Physicochemical characterization of CPMs and CPNMs. (A) IR spectra. (B) XRD patterns. (C) DSC thermograms. (1) raw CLZ, (2) PMs, (3) PNMs, (4) CPMs, (5) CPNMs, (6) physical mixture of CLZ and PMs, (7) physical mixture of CLZ and PNMs.

As shown in Figure 3A, the IR spectrum of CLZ shows the typical bands between 600 and 800 cm^−1^, which corresponds to the bending vibration of C—H bonds in aromatic conjugated rings. The —N—O stretching vibration appears at 1585 cm^−1^, and 1261 cm^−1^ for C—O stretching. The C—Cl stretching vibration observed at 765 cm^−1^ is of critical significance for the pharmacological activity of the drug.^42^ The typical absorption bands of PMs and PNMs are observed at 3436 cm^−1^ (O—H stretching), 2998, 2948, 2884 cm^−1^ (CH_2_, CH_3_ stretching), 1763 cm^−1^ (C=O stretching vibration), and the 1091–1275 cm^−1^ range (=C—O stretching), which indicated the particles were prepared from PLGA.^27^, ^34^ The IR spectra of CPMs and CPNMs showed that the original bands of CLZ in the range of 600–800 cm^−1^ disappeared, while the characteristic peak of PLGA at 1763 cm^−1^ for C=O stretching remained, which suggested that the drug was encapsulated in the inner part of CPMs and CPNMs. The typical characteristic peaks of CLZ and PLGA can be simultaneously observed in the physical mixture.

From the XRD patterns (Figure 3B), the sharp and narrow diffraction peaks for CLZ can be observed in the range of 2*θ* = 0°–60°, which is consistent with previous study.^29^ In the physical mixed group of raw CLZ, PMs, and PNMs, the same diffraction peaks are also observed in the range. However, no typical diffraction peaks of CLZ were observed for CPMs and CPNMs. In addition, as shown in Figure 3C, a melting point for raw CLZ was observed at 146.5°C, which disappeared in the CPM and CPNM traces.^8^ The *T*_*g*_ values of CPMs and CPNMs were measured at 45.17 and 45.67°C, which were lower than those of PMs and PNMs (47.35°C, 46.98°C).^34^ In the physical mixture groups, the melting point of CLZ was still observed, with higher *T*_*g*_ values of PLGA observed at 53.78 and 54.87°C.

### 3.2 In vitro release kinetics

**FIGURE 4:**
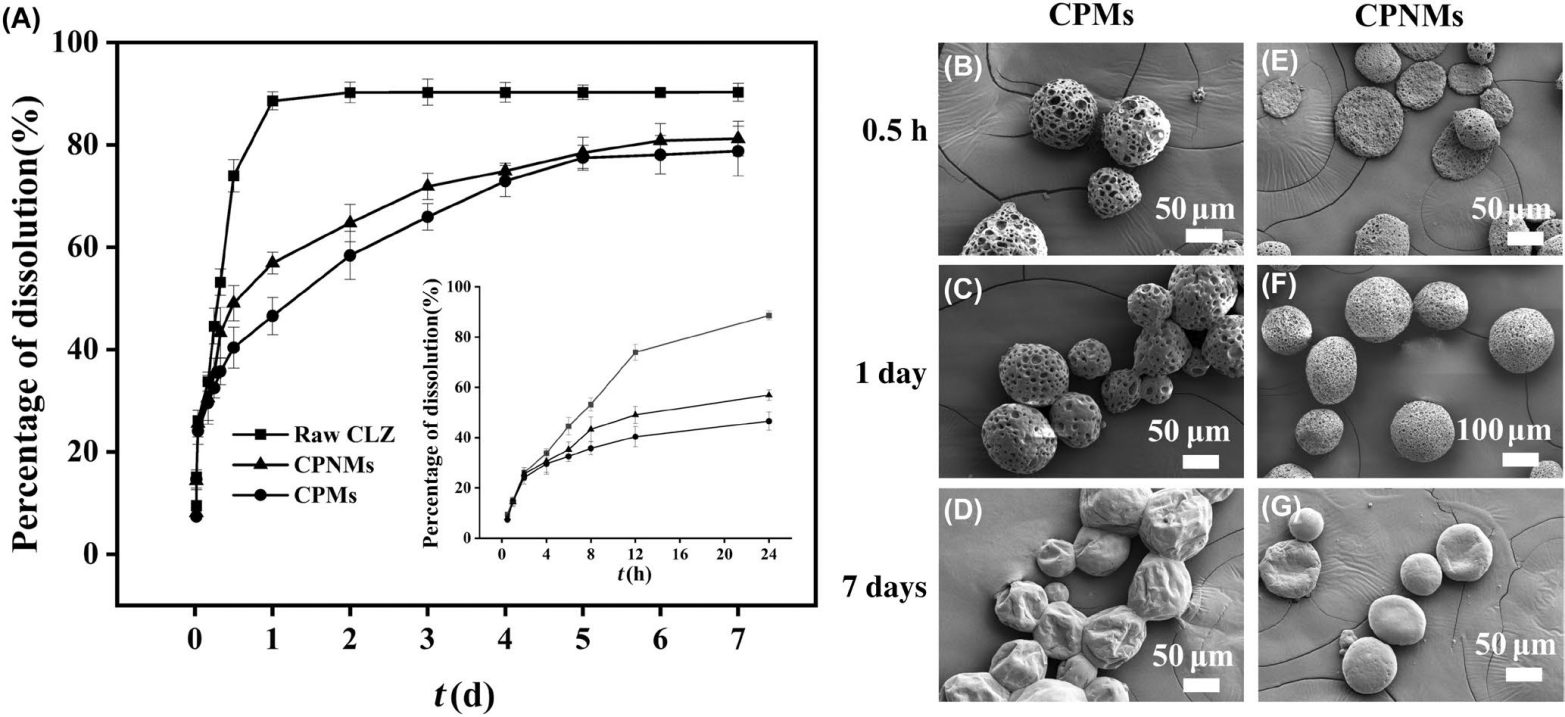
CLZ release profiles and SEM images of CPMs and CPNMs during the drug release study. (A) CLZ release profiles of raw CLZ (‐■‐), CPNMs (‐▲‐), and CPMs (‐●‐) (*n* = 3). SEM images of CPMs (B,C,D) and CPNMs (E,F,G) after drug release over different times.

As shown in Figure 4A, raw CLZ showed a rapid dissolution profile. It was rapidly dissolved in the first 12 h, followed by a deceleration in dissolution rate. By 1 day, it was almost completely dissolved, with a cumulative release of 88.57%. The dissolution process continued over 7 days, culminating in a final cumulative release of 90.29%. After 1 day, the cumulative release of CLZ in CPMs and CPNMs was 46.55% and 56.89%, respectively. After 7 days, the cumulative release of CLZ in CPMs was 78.78%, and that in CPNMs was 81.19%. The CLZ release profiles of CPMs and CPNMs were fitted to several release models (Zero order, First order, Higuchi, Weibull, Ritger‐peppas) (Table S1). It was found that the drug release profiles of both groups were more consistent with the Weibull equation. In the Ritger‐Peppas model, disc‐shaped particles (a type of thin polymer slab model) have a diffusion exponent (*n*) of 0.50 for Fickian diffusion and 1.00 for Case‐II transport, and the release exponents of CPMs and CPNMs were 0.39 and 0.36, indicating that CLZ release of both samples was controlled by Fickian diffusion.^27^, ^34^, ^41^

The SEM images of CPMs after CLZ release showed that the surface pore size of CPMs gradually decreased after 1 day, and the pores completely disappeared and the surface became smooth on day 3, showing the change from porous microspheres to solid microspheres. After 5 days, there was a depressed appearance on the surface of the microspheres, while the overall spherical structure was maintained. After 7 days, the surface of CPMs was wrinkled and collapsed, with adhesion at the edges among particles (Figures 4B–C and S2A–C). The original CPNMs were thin discs with a coarse surface. After CLZ release, the particles underwent swelling and the thickness of the non‐spherical particles increased after 1 day (Figures 4E,F and S2D,F). After 3 days, the CPNMs transformed into microspheres characterized by a smooth surface and the presence of pores (Figure S2E). By 5 days, the microparticles maintained dense surface porosity while exhibiting slight adhesion (Figure S2F). With an increase in CLZ release time, the particles swelled and then finally collapsed into smooth discs (Figure 4G).

### 3.3 Vaginal local pharmacokinetics

**FIGURE 5:**
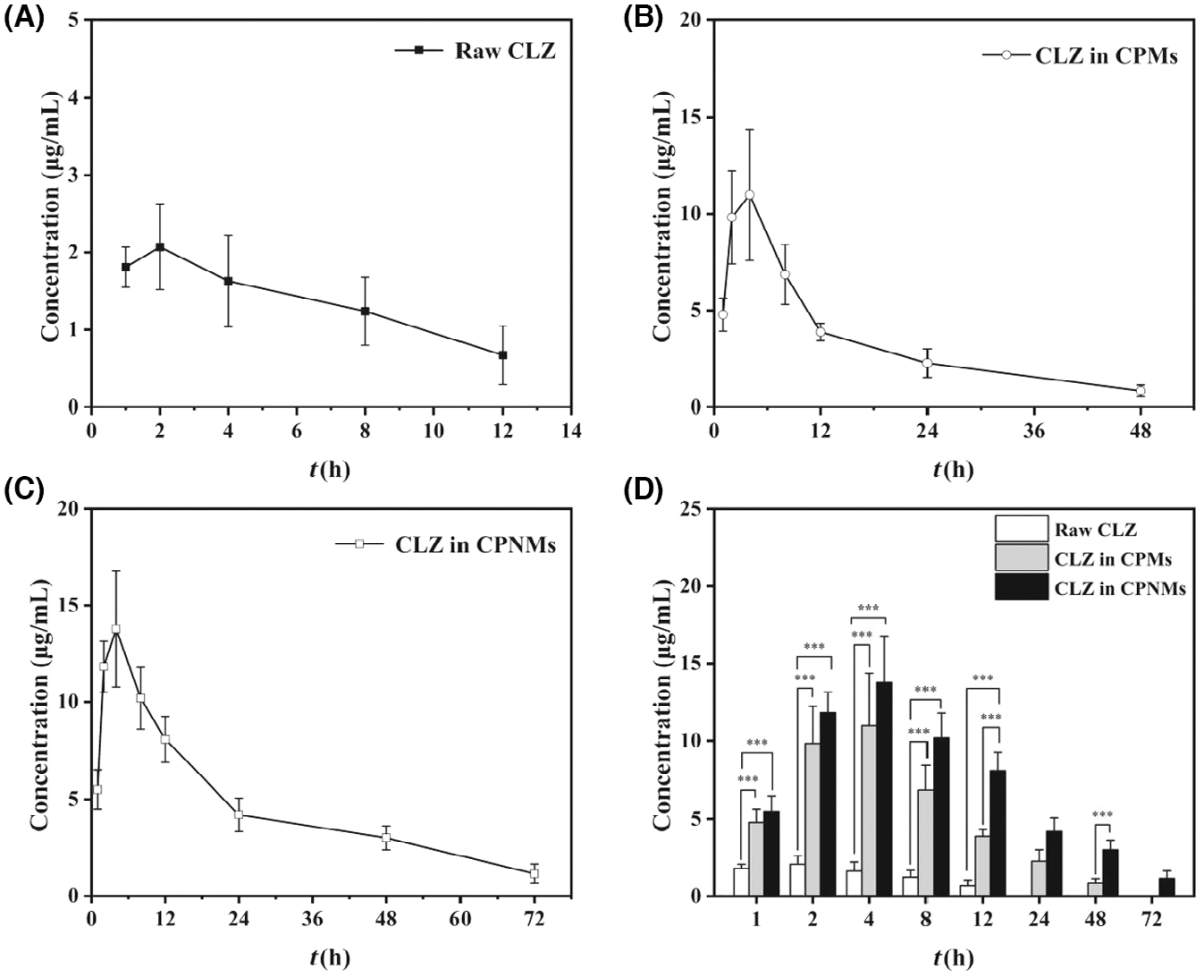
Effect of microparticles shape on the pharmacokinetics. CLZ concentrations in vaginal lavages following intravaginal administration of raw CLZ (A), CPMs (B) and CPNMs (C), and the corresponding histograms (D). Data are shown as mean ± SD, *n* = 4, ***p < 0.001.

Figure 5 showed the pharmacokinetic curves of raw CLZ, CPMs and CPNMs after a single intravaginal administration. Table S2 provides pharmacokinetic parameters of each group. The drug concentration of raw CLZ was significantly lower than that of CPMs and CPNMs, reaching a maximum level of 2.07 μg/mL at 2 h and 0.67 μg/mL after 12 h (Figure 5A). The drug concentrations in the vaginal lavage fluid of the CPM and CPNM groups reached their peaks at 4 h, with concentrations of 10.98 μg/mL and 13.77 μg/mL, respectively, which were approximately 5–7 times higher than those of raw CLZ, and vaginal drug levels began to decline gradually until 4 h (Figure 5B–D). The CLZ concentration in the CPM group reached its lowest level of 0.84 μg/mL at 48 h, which was still higher than that in the raw CLZ group. At 72 h, CLZ was detectable in the CPNM group (1.15 μg/mL) (Figure 5B,C).

### 3.4 In vitro blood compatibility

**FIGURE 6:**
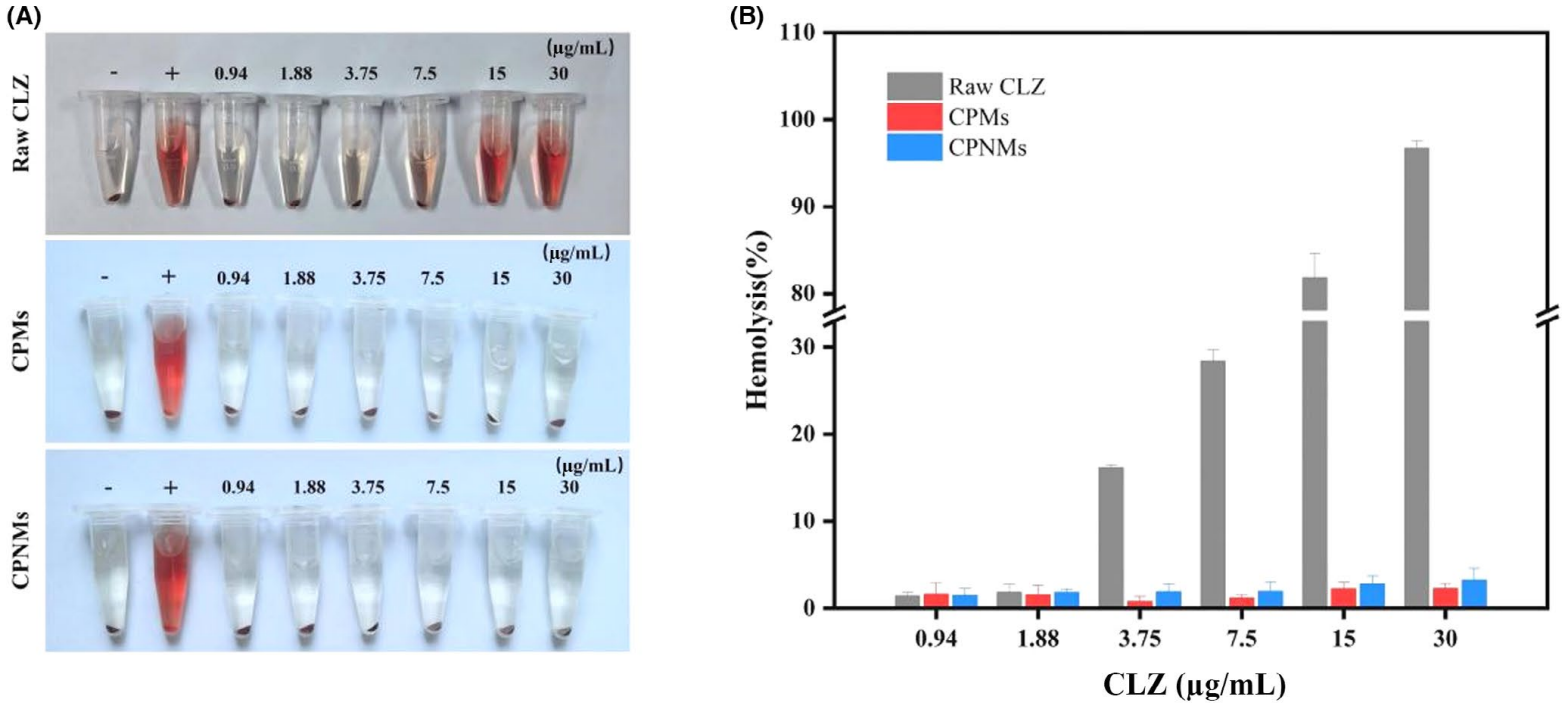
Effect of CPMs and CPNMs with varying CLZ concentrations on mice red blood cells. (A) Hemolysis images. (B) Hemolysis rates of raw CLZ, CPMs and CPNMs with varying CLZ concentrations (*n* = 3).

As shown in Figure 6A, the hemolysis of raw CLZ showed a dose‐dependent pattern, with the hemolysis rate increasing as the concentration of CLZ increased. At a CLZ concentration of 3.75 μg/mL, hemolysis was observed, with a hemolysis rate of 16.19%. In contrast, at the maximum concentration (30 μg/mL, CLZ), the hemolysis rates of the CPM and CPNM groups were 2.27% and 3.24%, respectively, indicating the absence of hemolytic activity (Figure 6B). These findings suggest that the cytotoxicity of CLZ can be reduced by CLZ encapsulation, and both CPMs and CPNMs exhibited good blood compatibility.

### 3.5 In vivo biocompatible evaluation

**FIGURE 7:**
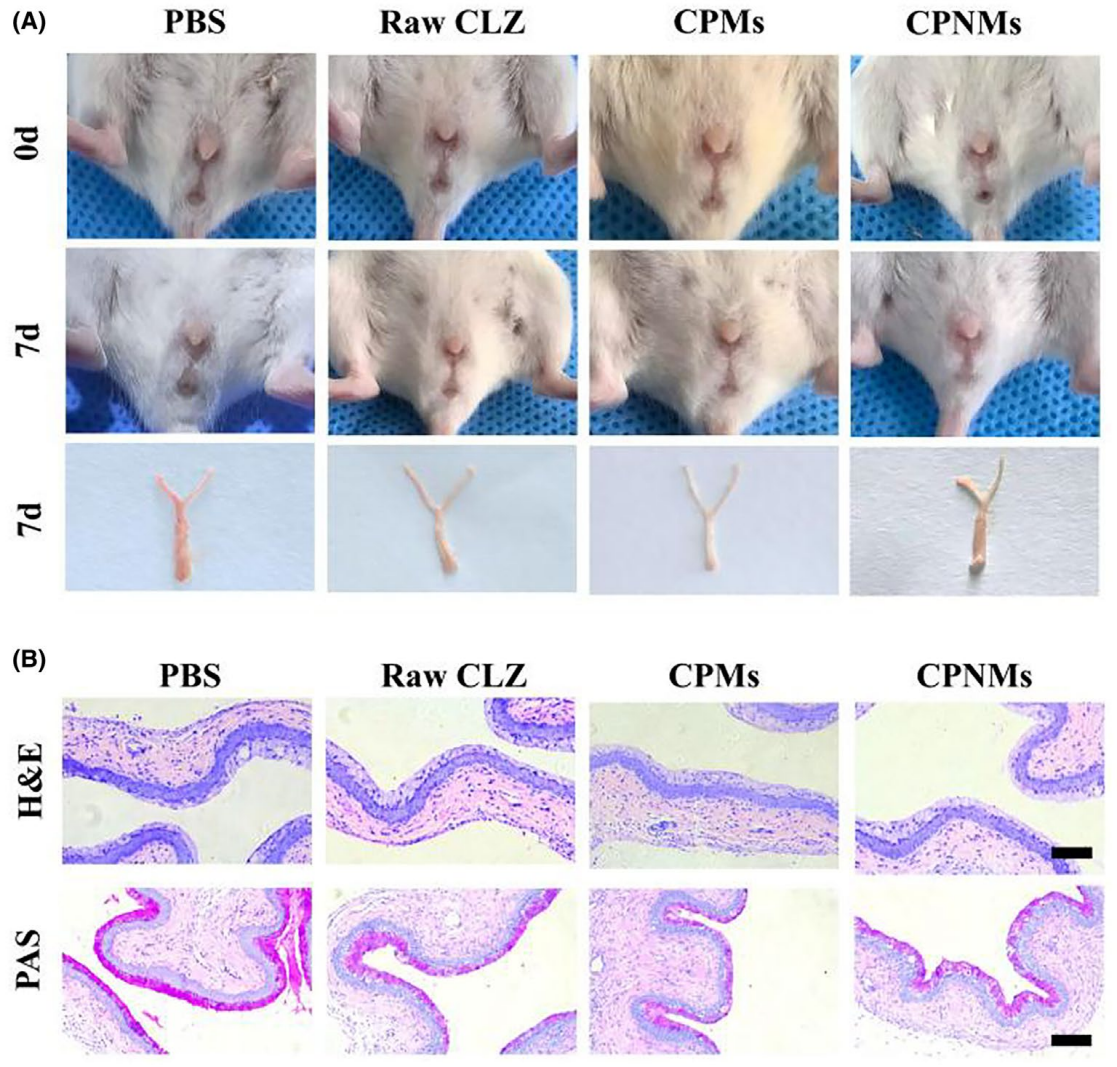
Irritation of vaginal mucosa by CPMs and CPNMs. Following 7 successive days of intravaginal injection with PBS, raw CLZ, CPMs and CPNMs with comparable dosages of CLZ (5 mg/kg), the vaginal conditions of healthy mice were assessed across the various groups by macroscopic observation (A) and histological analysis (H&E and PAS staining) (B). Scale bar = 100 μm (*n* = 6).

**FIGURE 8:**
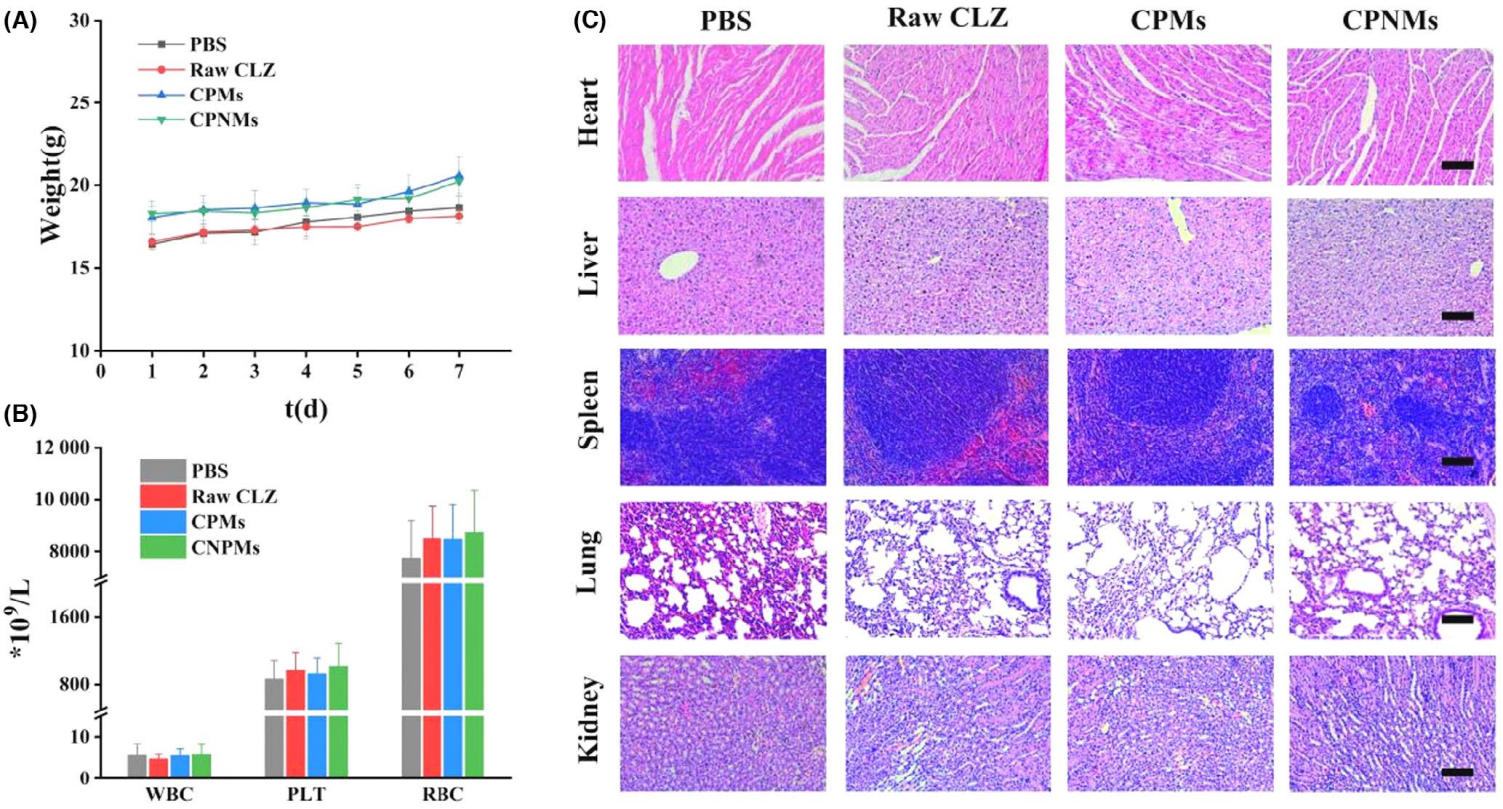
Biocompatible evaluation of CPMs and CPNMs in vivo. (A–C) Body weight (A), blood routine profiles (B) and histological analysis (C) of healthy mice after treatment with PBS, raw CLZ, CPMs and CPNMs at a comparable dosages of CLZ (5 mg/kg) per day for 7 consecutive days. WBC: White blood cells; PLT: Platelets; RBC: Red blood cells. Scale bar = 100 μm. Data are shown as mean ± SD, *n* = 6.

As shown in Figure 7A, the suspensions of raw CLZ, CPMs and CPNMs were administered in the vaginas of healthy mice, with CLZ dosed equivalently at 5 mg/kg for 7 days consecutively. There were no obvious irritating reactions such as hyperemia, erythema, swelling or ulceration on the vaginal of mice in each group. Histological analysis showed that the vaginal mucosal barrier was not destroyed in each group. The vaginal epithelium was intact, and both the cell morphology and glycogen secretion were normal, suggesting that raw CLZ, CPMs and CPNMs caused no obvious irritation and damage to the vaginal mucosa of mice (Figure 7B). The average body weight of mice in each group steadily increased during the administration period (Figure 8A), and no differences were observed in the major hematology‐related cells, including white blood cells (WBC), platelets (PLT), and red blood cells (RBC) (Figure 8B). Additionally, the major organs of the mice (heart, liver, spleen, lungs, and kidneys) showed no obvious abnormalities (Figure 8C). These results indicated that all the drug suspensions of raw CLZ, CPMs and CPNMs had no adverse effects on vaginal tissues and exhibited low systemic toxicity.

## 4 DISCUSSION

In the field of mucoadhesive strategies, it has been recognized that particles with coarse surfaces or non‐spherical shapes can prolong the residence time of drugs in the mucosa owing to their unique geometric shapes and surface textures, thereby promising potential benefits in vaginal drug delivery.^5^, ^23^, ^25^ In this study, we employed NH_4_HCO_3_ as a porogen to prepare CPMs with a pore structure. Subsequently, by varying the concentration of NH_4_HCO_3_ and the magnitude of shear force, we prepared CPNMs (Figures 1C and 2D). High concentrations of NH_4_HCO_3_ accelerated gas generation and reduced interfacial tension, thereby promoting the formation of hollow structures in W_1_/O emulsions. Under conditions of higher shear force, this process induces the collapse of porous microspheres, eventually leading to the formation of non‐spherical particles, as shown in our previous studies (Figure 2A–C).^34^, ^41^ As the CLZ concentration increased, the drug loading and the encapsulation efficiency of CPNMs were slightly lower than those of CPMs (Figures 1F and 2F). This difference is likely attributable to partial drug loss during the structural collapse of the hollow state induced by NH_4_HCO_3_.^27^

The results of physicochemical characterizations showed that the *T*_*g*_ values of CPMs and CPNMs (45.17°C, 45.67°C) were lower than those of PMs and PNMs (47.35°C, 46.98°C). The reductions may be attributed to CLZ as a plasticizer dispersed in PLGA.^27^, ^34^ However, the *T*_*g*_ values of PLGA in the physical mixture groups were higher than those of other groups, which can be attributed to the anti‐plasticization effect of CLZ.^34^ The IR spectra and XRD patterns of CPMs and CPNMs show that the original bands and the characteristic diffraction peaks of CLZ disappeared, which indicated that CLZ was successfully encapsulated into PLGA porous microspheres and PLGA non‐spherical particles, and might be dispersed as molecules in the PLGA matrix, irrespective of particle morphology (Figure 3A,B).^8^, ^34^, ^42^

When designing vaginal drug delivery systems, careful attention must be given to both the duration of the formulations in the vaginal cavity and the optimization of pharmaceutical release within desired timeframes.^8^, ^10^ This study further evaluated the effects of the shape and surface structure of the polymer particles on the in vitro release of CLZ. Compared with the raw CLZ, both CPMs and CPNMs had sustained release effects, with rapid release in the first 8 h for prompt treatment of VVC, followed by slow release for long‐term treatment. The release kinetics of CLZ from PLGA solid particles is governed by Fickian diffusion driven by a chemical potential gradient rather than by erosion, chemical reaction, or convective transport, whereby drug molecules spontaneously diffuse from regions of higher concentration to lower concentration through the porous structures of CPMs and CPNMs.^34^, ^43^ This release mechanism enables controlled drug release, characterized by an initial rapid release to achieve therapeutically relevant concentrations, followed by a decelerated release rate as the concentration gradient diminishes.^44^ The resulting sustained and prolonged release profile facilitates the optimization of dosing regimens, thereby improving patient compliance. The sustained release profile may represent a favorable factor in maintaining effective CLZ concentrations in the vagina over extended periods.^35^ During the in vitro drug release process, the morphologies of CPMs and CPNMs were observed at different time points. As shown in Figures 4B–G and S2, CPMs transformed from porous microspheres to regular microspheres and eventually exhibited adhesion. In contrast, CPNMs first underwent swelling, then transformed into porous microspheres, and finally collapsed into discoid particles with smooth surfaces. The shape of the PLGA non‐spherical particles changed from disc‐like to quasi‐spherical under the influence of alcohol, which can be ascribed to chemical additive‐responsive shape switching of polymer particles.^38^, ^45^, ^46^ Moreover, the hydrolysis of the PLGA microspheres under acidic release medium conditions generates acidic oligomers, which accelerated the drug release of CPMs and CPNMs. The release involved the matrix being eroded and the particles finally adhering to each other.^45^ Compared to CPMs, the swelling of CPNMs facilitated greater penetration of the release medium into the microparticle's interior. In addition, a longer duration was required for the surface pores of CPNMs to disappear, which also accelerated drug release. This might account for the slightly faster release of CLZ observed in CPNMs.^34^ Meanwhile, the hydrolysis of PLGA yields lactic and glycolic acid byproducts that contribute to preserving the natural acidic conditions of the female reproductive tract. The above results suggested that CPNMs showed ideal drug loading, and sustained‐release effects, laying a foundation for subsequent animal experiments.

To enhance local drug concentration while minimizing potential toxic effects, optimization of the vaginal drug delivery system is required, focusing on local distribution, absorption, and retention time.^19^, ^37^ Sodium hyaluronate can enhance the hydrophilicity of the particles and provide a lubricating effect of the particles.^36^ CPMs and CPNMs were uniformly dispersed in sodium hyaluronate solution, avoiding clogging of the needle and damage to the vaginal mucosa during administration.^27^, ^36^ After intravaginal administration, we observed that in the raw CLZ group, the drug concentration was only detectable for 12 h. Subsequently, CLZ was not detected in the vagina, which might be due to the easier removal of free drug by the vaginal self‐clearing mechanism.^5^ The vaginal drug concentration and retention time in the CPMs and CPNMs groups were significantly higher than those in the raw CLZ group (Figure 5). Compared to CPMs with a detection window of only 48 h, CPNMs extended observed intravaginal retention to 72 h. CPNMs enabled the maintenance of drug levels for a longer duration, with concentrations exceeding those of the raw CLZ and CPMs groups at each time point. The explanation for this is that the flat and coarse morphology of CPNMs can be better dispersed in the vaginal cavity, giving an enhanced contact area with the vaginal mucosa.^25^, ^27^ This study showed that although CPNMs could not directly penetrate the cervicovaginal mucus, the coarse surface and non‐spherical shape of the particles significantly prolonged retention time in the vaginal cavity to over 72 h in comparison with both raw CLZ and porous spherical counterparts.^26^, ^27^ This prolonged retention facilitated the sustained drug release of CLZ, allowing it to permeate the vaginal epithelium from the drug reservoir, thereby potentially effectively eliminating *Candida albicans*.

To assess the biosafety of CPNMs, a high dosage (comparable to 5 mg/kg of CLZ) was intravaginally administered to mice for 7 days consecutively.^20^ No signs of inflammatory irritation were observed in the vaginal appearances. Histological analysis revealed normal morphology of vaginal epithelial cells and regular glycogen secretion, with no abnormalities detected in tissues including the heart, liver, spleen, lungs, and kidneys. These findings indicated that CPNMs exhibited good biocompatibility.

## 5 CONCLUSION

In summary, we prepared CPNMs and CPMs by the double emulsion‐solvent evaporation method via adjustment of shear force and NH_4_HCO_3_ concentration. In comparison to porous spherical CPMs, the disc‐like shape and coarse surface of CPNMs enhanced their adhesion to vaginal mucosa and resistance to the self‐cleansing action of vagina. The local pharmacokinetics in the vagina further verified that CPNMs had a sustained and higher CLZ concentration in vaginal lavages due to the shape effect, which enhanced the therapeutic efficiency of CLZ against VVC. In addition, CPNMs showed low toxicity to vaginal epithelial cells, preserved the integrity of the vaginal mucosal barrier, and exhibited excellent biocompatibility, indicating the potential application of CPNMs in vaginal delivery. Overall, PLGA non‐spherical microparticle‐based drug delivery systems open a new avenue for managing vaginal infections and other women's health‐related conditions. Further investigations should focus on evaluating the pharmacodynamics of this drug delivery system in VVC models.

## AUTHOR CONTRIBUTIONS

**Yanyu Li:** Data curation; formal analysis; investigation; methodology; writing – original draft. **Jingting Luo:** Data curation; formal analysis; investigation; methodology; validation. **Shitao Zou:** Formal analysis; investigation; methodology; validation. **Xin Zhang:** Investigation; methodology; validation. **Zhen Wang:** Investigation; methodology. **Ziwen Jiang:** Conceptualization; supervision; writing – review and editing. **Jingjie Wang:** Conceptualization; methodology; supervision; writing – review and editing. **Zhaoxia Liu:** Conceptualization; funding acquisition; supervision; writing – review and editing. **Zhimin Zhou:** Conceptualization; funding acquisition; project administration; supervision; writing – review and editing.

## FUNDING INFORMATION

This work was supported by the Tianjin Natural Science Foundation for Beijing‐Tianjin‐Hebei Collaboration (23JCZXJC00240), Hebei Natural Science Foundation (H2023201903), Beijing Natural Science Foundation (J230006), and the CAMS Innovation Fund for Medical Sciences (2021‐I2M‐1‐052), the Opening Funding of Hubei Key Laboratory of Natural Products Research and Development, China Three Gorges University (2025NPRD2‐04).

## CONFLICT OF INTEREST STATEMENT

Zhimin Zhou is an editorial board member of Animal Models and Experimental Medicine (*AMEM*) and a corresponding author of this article. To minimize bias, he was excluded from all editorial decision making related to the acceptance of this article for publication.

## ETHICS STATEMENT

The experimental procedures involving these animals received formal approval from the Animal Ethical and Welfare Committee of the Experimental Animal Center of the Institution of Radiation Medicine, Chinese Academy of Medical Sciences (approval number: IRM/2‐IACUC‐2506‐033).

## Supporting information


Figure S1

## Data Availability

Date will be made available on request.
