# Supplementary material for: Clotrimazole‐loaded PLGA microparticles for local drug delivery to the vagina: Shape does matter
Source: Animal Model Exp Med. 2026 Apr 6;9(4):752–64. doi: 10.1002/ame2.70198 (PMC13242732; doi:10.1002/ame2.70198)
Supplement: Supplementary file 1 — Figure S1 [file AME2-9-752-s001.docx]

**Clotrimazole-loaded PLGA microparticles for local drug delivery to the vagina: shape does matter**

Yanyu Li^1,2^, Jingting Luo^2^, Shitao Zou^1,2^, Xin Zhang^3^, Zhen Wang^3^, Ziwen Jiang^4,*^, Jingjie Wang^2,5^, Zhaoxia Liu^1,2*^, Zhimin Zhou^2,3*^

^1^Hubei Key Laboratory of Natural Products Research and Development, College of Biological and Pharmaceutical Sciences, China Three Gorges University, Yichang 443002, China

^2^College of Biological and Pharmaceutical Sciences, China Three Gorges University, Yichang 443002, China

^3^Biomedical Barriers Research Center, Institute of Biomedical Engineering, Chinese Academy of Medical Sciences & Peking Union Medical College, Tianjin 300192, China

^4^Department of Gynecology, Beijing Obstetrics and Gynecology Hospital, Capital Medical University, Beijing Maternal and Child Health Care Hospital, Beijing 100006, China

^5^State Key Laboratory of Eye Health, Eye Hospital, Wenzhou Medical University, Wenzhou 325027, China

*Corresponding authors:

Zhimin Zhou

E-mail: zhouzm@bme.cams.cn

ORCID: 0000-0003-4832-3530

Zhaoxia Liu

E-mail: 27976574@qq.com

Ziwen Jiang

E-mail: jiangziwen@ccmu.edu.cn


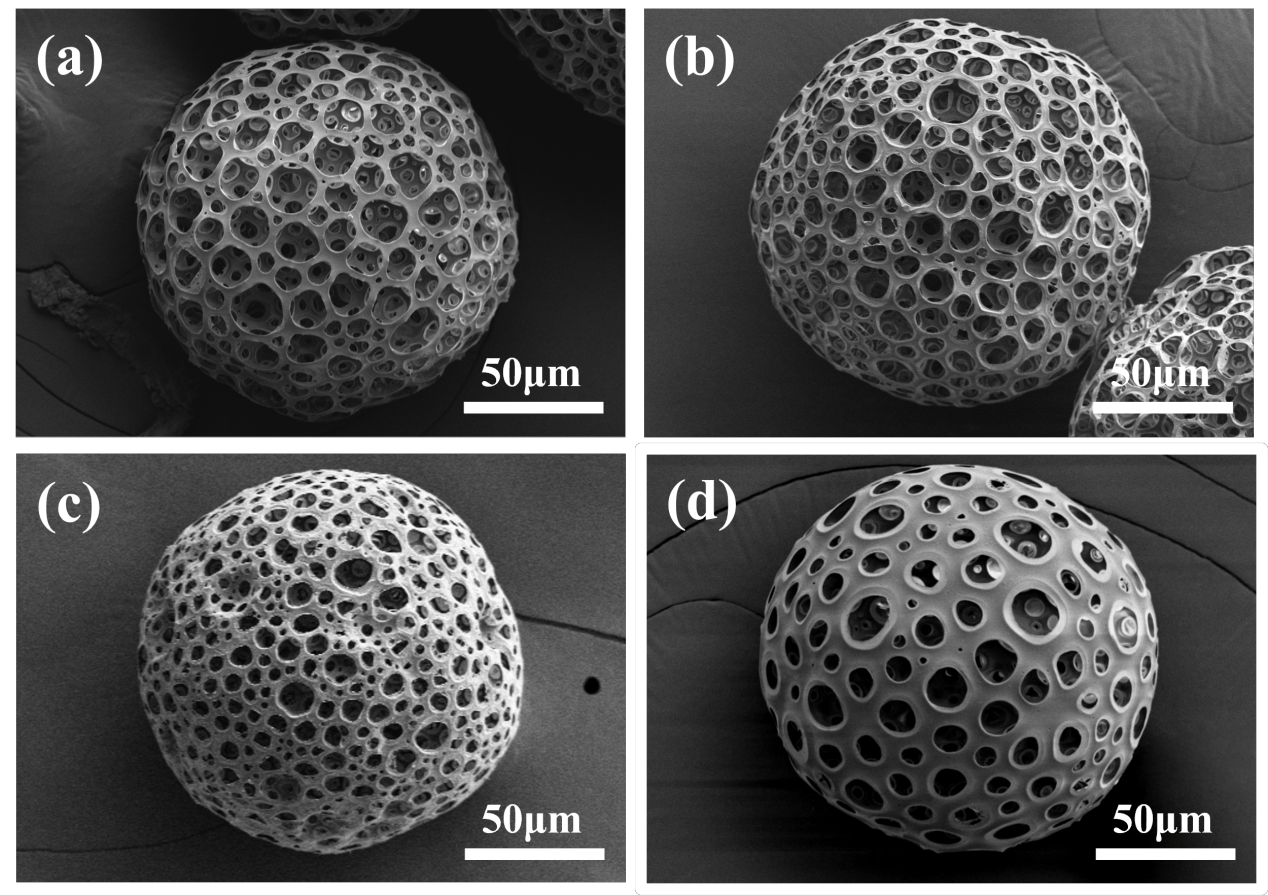


**Figure S1** SEM images of different CPMs with varying CLZ concentrations. (a) 1.25 mg/mL; (b) 2.5 mg/mL; (c) 3.75 mg/mL; (d) 5 mg/mL


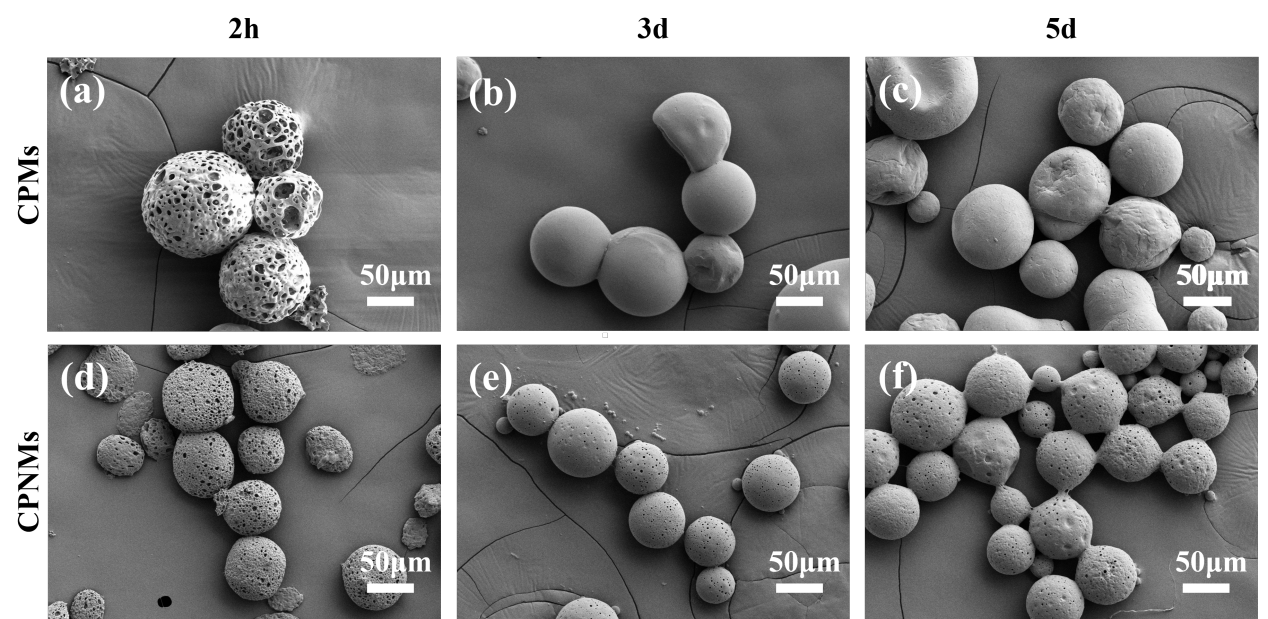


**Figure S2** SEM images of CPMs (a-c) and CPNMs (d-f) after drug release over different time.

**Table S1** *In vitro* dissolution models.

|  |  | Equation | Regression Coefficient (R^2^) |
| --- | --- | --- | --- |
| CPMs | Zero order | y=10.015+0.577t | 0.816 |
|  | First order | y=68.539(1-e^-0.135t^) | 0.931 |
|  | Higuchi | y=6.993t^0.5^+3.893 | 0.944 |
|  | Weibull | y=182.225(1-e^-(0.001(t-0.459))0.321^) | 0.995 |
|  | Ritger-Peppas | y=12.019t^0.392^  n=0.392±0.021 | 0.967 |
| CPNMs | Zero order | y=14.451+0.571t | 0.798 |
|  | First order | y=71.979(1-e^-0.143t^) | 0.963 |
|  | Higuchi | y=7.051t^0.5^+6.348 | 0.935 |
|  | Weibull | y=87.603(1-e^-(0.046(t-0.376)0.455^) | 0.999 |
|  | Ritger-Peppas | y=14.923t^0.355^  n=0.355±0.026 | 0.965 |

**Table S2** Pharmacokinetic parameters of CLZ in vaginal lavage fluid of mice after intravaginal administration.

| Parameters (Unit) | Raw CLZ | CPMs | CPNMs |
| --- | --- | --- | --- |
| AUC (mg/L*h) | 16.11 | 160.70 | 331.41 |
| MRT (h) | 5.17 | 13.82 | 22.88 |
| T max (h) | 2 | 4 | 4 |
| C_max_ (mg/L) | 2.07 | 10.98 | 13.77 |
| C_max_/AUC | 0.13 | 0.07 | 0.04 |
